# Natural Variation for Lifespan and Stress Response in the Nematode *Caenorhabditis remanei*

**DOI:** 10.1371/journal.pone.0058212

**Published:** 2013-04-26

**Authors:** Rose M. Reynolds, Patrick C. Phillips

**Affiliations:** Institute of Ecology and Evolution, University of Oregon, Eugene, Oregon, United States of America; University of Washington, United States of America

## Abstract

Genetic approaches (*e.g.* mutation, RNA interference) in model organisms, particularly the nematode *Caenorhabditis elegans*, have yielded a wealth of information on cellular processes that can influence lifespan. Although longevity mutants discovered in the lab are instructive of cellular physiology, lab studies might miss important genes that influence health and longevity in the wild. *C. elegans* has relatively low natural genetic variation and high levels of linkage disequilibrium, and thus is not optimal for studying natural variation in longevity. In contrast, its close relative *C. remanei* possesses very high levels of molecular genetic variation and low levels of linkage disequilibrium. To determine whether *C. remanei* may be a good model system for the study of natural genetic variation in aging, we evaluated levels of quantitative genetic variation for longevity and resistance to oxidative, heat and UV stress. Heritability (and the coefficient of additive genetic variation) was high for oxidative and heat stress resistance, low (but significant) for longevity, and essentially zero for UV stress response. Our results suggest that *C. remanei* may be a powerful system for studying natural genetic variation for longevity and oxidative and heat stress response, as well as an informative model for the study of functional relationships between longevity and stress response.

## Introduction

Current understanding of the genetic basis of the aging process has been formed mainly by mutagenesis and RNA interference studies in the model systems *Saccharomyces cerevisiae* (yeast), *Caenorhabditis elegans* (soil-dwelling roundworm), and *Drosophila melanogaster* (common fruit fly) [1]. Most loci identified as affecting lifespan in those species are members of evolutionarily conserved metabolic pathways such as the insulin/insulin-like signaling [2], target-of-rapamycin [3], Jun kinase [4] and sirtuin [5] pathways. “Age mutants” can be defined as those displaying extreme longevity, falling well above the maximum lifespan observed under normal laboratory conditions. Although natural populations do not display the extreme longevity achieved by Age mutants, quantitative genetic research has revealed that there is significant and heritable variation in lifespan within genetically heterogeneous populations, *e.g. h^2^* = 0.03 [6] to *h^2^* = 0.73 [7]. This implies that studies of the underlying genetics of aging in natural populations may suggest promising molecular targets for intervention in age-related disease and dysfunction.

*C. elegans* has been a workhorse in the genetics of aging: mutagenesis and gene expression studies in *C. elegans* have facilitated a detailed understanding of many longevity genes and their roles within key metabolic pathways [8]. Its genome has been sequenced and assembled, it has very short generation times (3–4 days), and it can be cultured at extremely large population sizes, facilitating the construction of genetic lines and the conduction of selection experiments. One notable advantage of *C. elegans* over *D. melanogaster* is that it can be frozen, allowing parental and descendant generations to be tested at the same time and allowing large numbers of lines to be maintained at minimal effort. Despite these advantages, the *C. elegans* model is not optimal for studies of natural genetic variation. Recently several studies have shown that *C. elegans* populations possess low levels of molecular genetic variation on a worldwide scale [9]–[11]. Worse, nearly all of the existing variation is locked within genome-wide linkage disequilibrium, yielding a small number of effective haplotypes [11]–[13]. Few studies have estimated heritability of lifespan in *C. elegans*, but published estimates are not significantly different from zero [14]. These issues suggest that *C. elegans* will serve as a poor model for studying variation in aging within natural populations [15].

In contrast, a close relative of *C. elegans*, *C. remanei*, may be an ideal system in which to study natural genetic variation. Unlike the low genetic variation found within the selfing *C. elegans,* populations of *C. remanei* harbor significant amounts of molecular genetic variation [16], [17], and linkage disequilibrium breaks down on the order of 100 base pairs [18]. Unlike the androdioecious *C. elegans*, *C. remanei* is composed of males and females, making it a better model for human aging. It is a non-parasitic, soil-dwelling roundworm that is easily collected in the wild. Significantly, it can be frozen within just a few generations of collection, thereby maintaining a majority of the natural genetic variation present in the population when in its natural habitat. Like *C. elegans*, its genome has been sequenced, it has short generation times (∼4 days), can be cultured at large population sizes, can be genetically transformed, and can be frozen, giving us the ability to compare evolved or manipulated genetic lines to the original ancestral population.

Given the large amount of molecular genetic variation previously found in *C. remanei* for a variety of molecular pathways [17], we predicted that *C. remanei* might possess the genetic variation for longevity and stress resistance necessary to make it a good model system for the study of natural variation in aging. Specifically, we looked for evidence of significant natural genetic variation for longevity and acute heat, oxidative and UV stress resistance in a genetically heterogeneous population of *C. remanei*. We found high heritability for oxidative and heat stress resistance, low but significant heritability for lifespan, and no heritability for UV stress resistance. These results suggest that *C. remanei* may be a good model in which to look for natural genetic variants increasing health and lifespan. In particular, it appears promising for investigations into the complex relationship between stress response and longevity.

## Materials and Methods

### Strains & Handling

Wild *C. remanei* were isolated from terrestrial isopods (woodlice) from a single population near Dayton, Ohio and generously supplied to us by Scott Baird (Wright State University) with the permission of the State of Ohio Department of Natural Resources (see [19] for details). Isofemale strains were created from mating pairs and inbred for 10 generations to create distinct genetic strains. Those strains were inbred via full-sib mating, and then individually frozen for long-term storage. We obtained nineteen of those strains (PB strains 234, 237, 241, 244, 245, 247, 254, 255, 256, 257, 258, 259, 261, 263, 266, 269, 271, 272, 285), added them in equal proportions to the same Petri plate with media, and allowed them to randomly mate for four generations in order to generate a genetically heterogeneous *C. remanei* population. This genetically heterogeneous population (PX323) was frozen at −80°C for future use (as were all strains used in this study). To measure heritable genetic variation for longevity and traits that may be correlated with longevity, we employed half-sib, strain-based, and parent-offspring regression approaches to measure genetic variation and estimate trait- and sex-specific heritability for various traits. All strains were thawed and given two generations to recover from freeze before collecting experimental individuals. Strains were maintained on NGM-Lite agar plates seeded with *Escherichia coli* (OP50) as a food source. All populations were housed in an incubator at 20°C after thaw and throughout the experiment.

#### Inbred Strain Design

To obtain stage-synchronized individuals for inbred strain-based measures of genetic variation, we randomly collected one female and one male fourth stage larvae (“L4,” virgin) from each of ten isofemale strains (PB lines 234, 241, 244, 245, 261, 266, 269, 271, 272). These mating pairs were given 24 hours to sexually mature and mate. Females were then moved to individual plates and allowed to lay eggs for 6–24 hours, after which all adults were discarded. Offspring were collected as L4s within a single six-hour window, and placed on seeded plates in groups of 10–15 overnight. 20 to 24 hours later the offspring had become young adults and were then used in either a heat shock or a longevity assay as described below.

#### Half-sib Design

For each assay, ten males (sires) were collected as L4s from the genetically heterogeneous PX323 stock. We housed each sire on his own small (35×10 mm) plate with three to five randomly selected L4 females (dams) from the same stock. Each sire was given 24 hours to sexually mature and mate with dams. We then placed each dam on her own small plate and allowed her to lay eggs for 24 hours, after which she was discarded. Because each dam mated with only one sire, all offspring from a single dam were full sibs; offspring from different dams mated to the same male were half-sibs. From each dam, we collected eight to 16 full-sib L4 male and female offspring and placed them on seeded NGM plates in same-sex groups overnight. 20 to 24 hours later those offspring had become young adults and were then used in either an oxidative or UV shock assay as described below. For each sire, the offspring from three separate dams were tested (3 half-sib families).

#### Parent-Offspring Design

We randomly collected 120 L4 females and 360 male L4s (grandparental generation) from the genetically heterogeneous PX323 stock. Each female was placed with three males. These mating groups were given 24 hours to sexually mature and mate, after which all adults were discarded. Female offspring (dams) were collected as L4s within a single six-hour window. Each dam was given three L4 sires (randomly selected from the PX323 stock) with which to mate. 24 hours later sires were discarded and dams were moved to new plates daily until reproduction had ceased, then once per week until death by natural causes. Three daughters were collected per female. Each L4 daughter was given three L4 males (randomly selected from the PX323 stock) with which to mate. 24 hours later, males were discarded and daughters were moved to new plates daily until reproduction had ceased, and then once per week until death by natural causes. Under this design, daughter lifespans (daughters could be full or half sibs) were regressed against dam lifespan.

### Phenotyping & Analysis

#### Longevity

We estimated genetic variation for longevity using both strain-based and parent-offspring regression methods. For both methods we maintained worms individually on small NGM-Lite plates that were seeded with OP50. All worms were transferred to freshly seeded plates at weekly intervals. Longevity was determined by checking each individual daily at the same time of day. Worms were declared dead if they did not respond to being prodded with a platinum wire.

We performed statistical analyses using JMP 8.0 and SAS 9.2. [20]. We determined the significance of strain-specific differences in mortality using the Cox proportional hazards model with main effects of strain and replicate within strain (not all strains were in all replicates). When estimating genetic variance for mean lifespan, individuals who had left the agar plates or been accidentally killed (right censored) were removed from the data set and genetic variance was determined using an analysis of variance including the random effects of strain and replicate within strain. Heritability was calculated as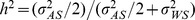, where 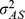 is the among-strain variance component and 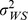 is the within-strain variance component [21]. Average *N* per strain: females = 35.9, males = 28.9. To confirm our strain-based estimate of heritability we performed a parent-offspring regression on the data collected as described above. We performed a general linear regression of daughter lifespan against parent (dam) lifespan. All lifespan data were log_10_ transformed to normalize residuals, and outliers were removed, *N* = 73 families. Standard errors on the estimates and significance tests were conducted via bootstrap analysis [22].

#### Heat Stress Resistance

We performed extensive preliminary testing to determine a temperature and length of exposure such that the genetically heterogeneous strain (PX323, a proxy for mean isofemale line response) experienced approximately 50% survival. We observed that thermal maximum for males is at least one full degree Celsius lower than that for females. Within sex, a change in temperature of half a degree made the difference between 80% and 0% survival (unpublished data). Therefore, we chose to test only females at this time.

L4 females were collected from ten of the nineteen isofemale strains (PB strains 234, 237, 241, 244, 245, 255, 258, 269, 271 and 272; strains were selected at random) as described above, and housed in same-sex groups of 10–12 on small, seeded NGM plates for 24–30 hours. We then placed them in strain-specific groups of 60 one-day-old virgin adults onto large, seeded NGM plates and exposed them to 35.5°C for 16 hours. We then moved the worms to 20°C and allowed them to recover for three hours before scoring plates for live and dead worms. Genetic variance for heat stress resistance ability was determined using an analysis of variance with random effects of strain and replicate. Heritability was calculated as for lifespan. Data were square root transformed to meet distribution assumptions for the analysis of variance. Average replication (*N*) per strain = 8. Three of ten strains had fewer than six replicates and were therefore excluded from the data set during analysis.

#### Oxidative Stress Resistance

Preliminary tests suggested that moderate differences in resistance among PX323 families would be detectable if oxidant-induced mortality caused 100% death between four and eight hours. As for heat stress resistance, males were less resistant to hydrogen peroxide than were females, but the difference in resistance between sexes was not extreme, therefore we tested both sexes using a single concentration and duration of exposure to hydrogen peroxide.

We placed one-day-old virgin adults into either 40 µL of 3.5 mM H_2_O_2_ (experimental individuals) or 40 µL of S-basal (controls). Worms were checked every 30 minutes until all experimental individuals failed to respond to prodding with a platinum wire for three continuous samplings (scored dead for 90 minutes). Estimates of genetic variance for oxidative stress resistance ability was determined using analysis of variance model with random effects of sire, dam within sire, and replicate. Heritability was calculated as 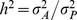, where 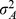 is additive genetic variance and 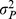 is total phenotypic variance [23].

#### UV Stress Resistance

Preliminary tests suggested that moderate differences in resistance among PX323 families would be detectable if UV exposure-induced mortality caused 100% death within one to two weeks. As for oxidative stress resistance, males were less resistant to UV stress than were females, but the difference in resistance between sexes was not extreme, therefore we tested both sexes using a single duration of exposure to UV irradiation.

We placed one-day-old adults with their same-sex full sibs onto large unseeded plates and exposed to 2000 J/m^2^ UV irradiation (FisherBiotech Microprocessor-Controlled UV Crosslinker FB-UVXL-1000 using 254 nm UV tubes). Worms were immediately picked to large seeded plates and checked daily until all worms failed to respond to prodding with a platinum wire. Estimates of genetic variance for UV stress resistance ability was determined using analysis of variance with random effect of sire, dam within sire, and replicate. Heritability was calculated as for oxidative stress response.

#### Calculation of CV_A_ and errors

For all traits, the coefficient of additive genetic variance (*CV_A_*) was calculated as 
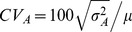
, where 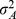 is additive genetic variance and *μ* is the mean of the phenotypic response [24]. Errors on quantitative genetic parameters were calculated as described in [25] where possible, and otherwise by bootstrapping [22]. Comparison of *h^2^* values was accomplished by bootstrapping the data and evaluating the extent of the overlap of the resulting distributions was via a Mann-Whitney U test.

## Results

**Figure 1:**
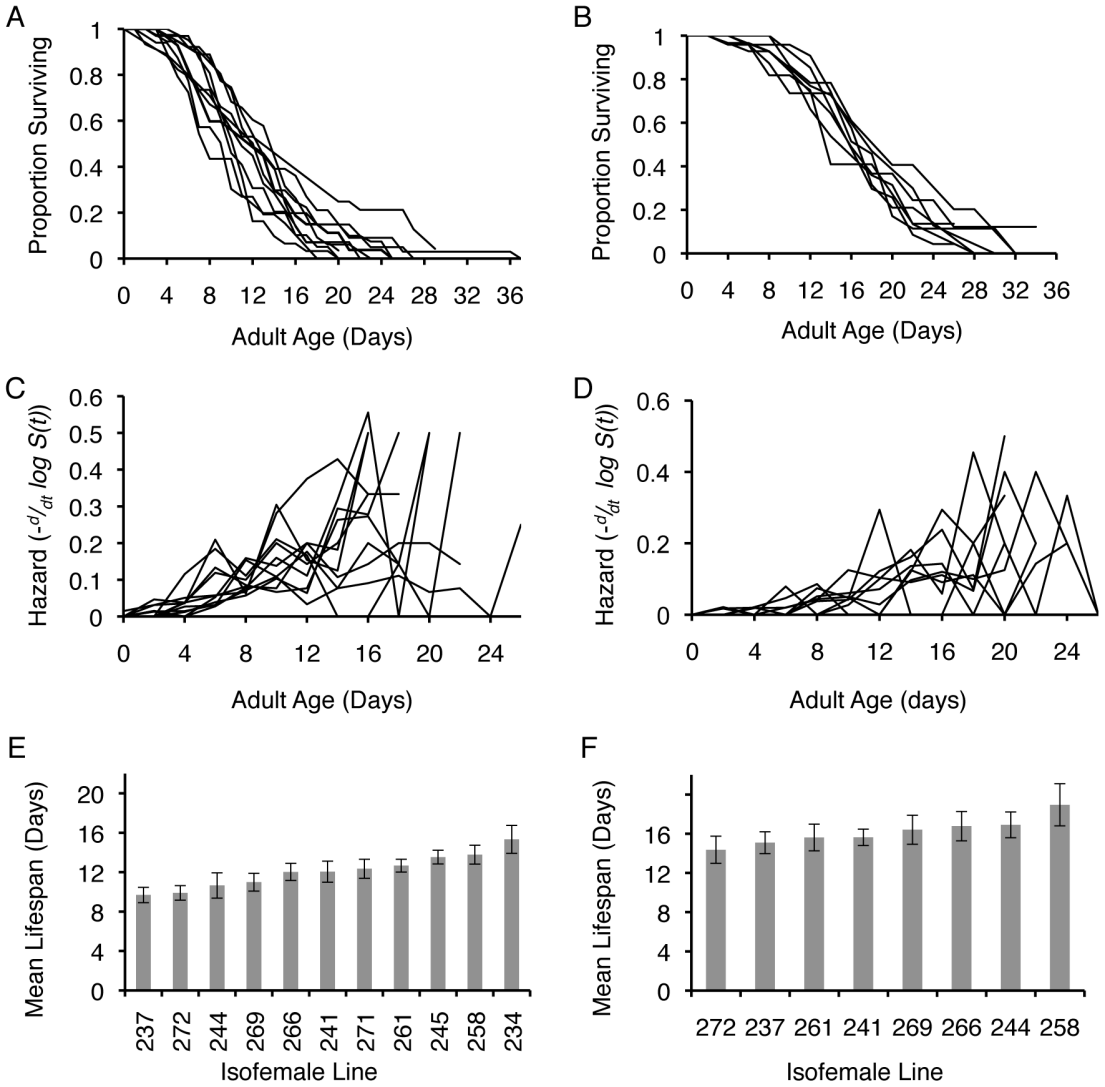
Differences in lifespan of *C.* remanei isofemale lines. Top row: age-specific survival (*S(t)*) of females (A), males (B). Middle row: age-specific hazard (risk of death) of females (C), males (D) Hazard is defined as the instantaneous risk of death. Although hazards have no universal scale, max hazard (100% risk of death within the following day) for these data is one. The reciprocal of the hazard 1/*h*(*t*) is expected time to death, given survival to age *t.* Bottom row: mean adult lifespan of females (E) and males (F); bars are standard errors. Lifespan displayed low but significant heritability and coefficient of additive genetic variation (Table 1).

Mean lifespan significantly differed among lines (analysis of variance; Females *F*_10, 280_ = 4.60, *P<*0.0001; Males *F*_10, 280_ = 4.60, *P<*0.0001; Figure 1a,b; Table S1), as did the age-specific risk of death (Cox proportional hazard; Females 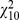 = 83.04, *P<*0.0001; Males 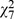
_ = _25.76, *P* = 0.0006; Figure 1C & D). For females, *CV_A_* and *h^2^* – measures of the additive genetic component of the differences in lifespan among lines – were small, but significantly greater than zero (Table 1), and within the range of published estimates for other organisms [6]. In contrast, for males neither *CV_A_* nor the line-based or parent-offspring estimates of *h^2^* for lifespan were significantly from zero (Table 1; Figure S1).

**Table 1:** Estimates of heritabilities and coefficients of additive genetic variation in *C. remanei.*

Trait	Method of estimation	Sex	h^2^(SE)	CV_A_
Lifespan	Inbred Lines	Female	0.08(0.04)**	13.07**
	Inbred Lines	Male	0.06(0.05)	12.85
	Parent-Offspring	Female	0.06(0.29)	9.14
Heat Stress Resistance	Inbred Lines	Female	0.45(0.18)*	28.59*
		Male	– 1	– 1
Oxidative Stress Resistance	Half-Sib	Female	0.95(0.55)**	22.05**
		Male	0.75(0.52)*	17.28*
UV Stress Resistance	Half-Sib	Female	−0.05(0.12)	0 2
		Male	−0.21(0.18)	0 2

* P<0.05, **P<0.01

1 Males not assayed.

2 Negative variance component estimates.

**Figure 2:**
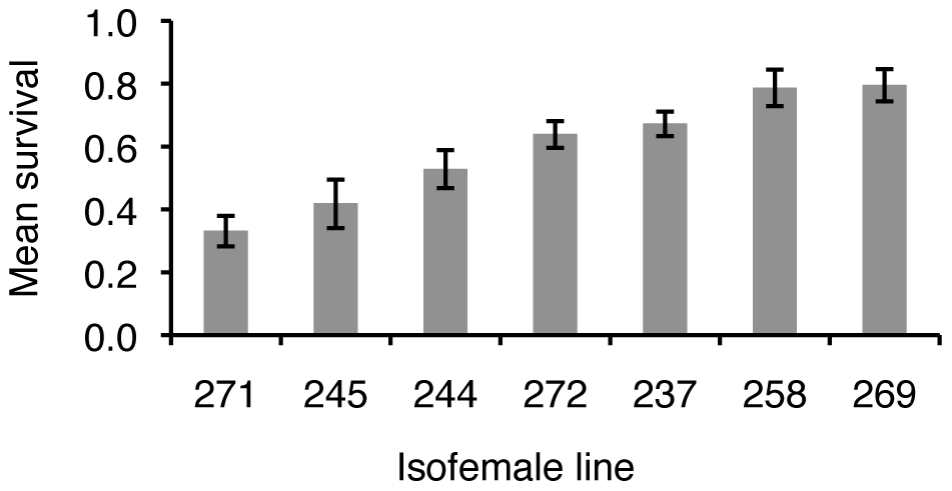
Mean survival of females from *C.*remanei isofemale lines after a 16 hour heat stress at 37.5°C. Worms were exposed as one-day-old adult virgins. Heritability for heat stress resistance was high (*h^2^* ^= ^0.45, Table 1), and significantly greater than those for longevity or UV stress (Mann-Whitney U, *P*<0.0001).

The ability to withstand heat stress differed significantly among lines (Females *F*_6,6_ = 12.06, *P* = 0.004; Figure 2; Table S2) and showed significant heritability in our population. Indeed, *h^2^* for heat stress resistance was more than five times greater than that for lifespan (Mann-Whitney U, *P*<0.0001, Table 1). This pattern was further reflected when comparing *CV_A_* between the two traits: *CV_A_* for heat stress was nearly twice that for longevity.

**Figure 3:**
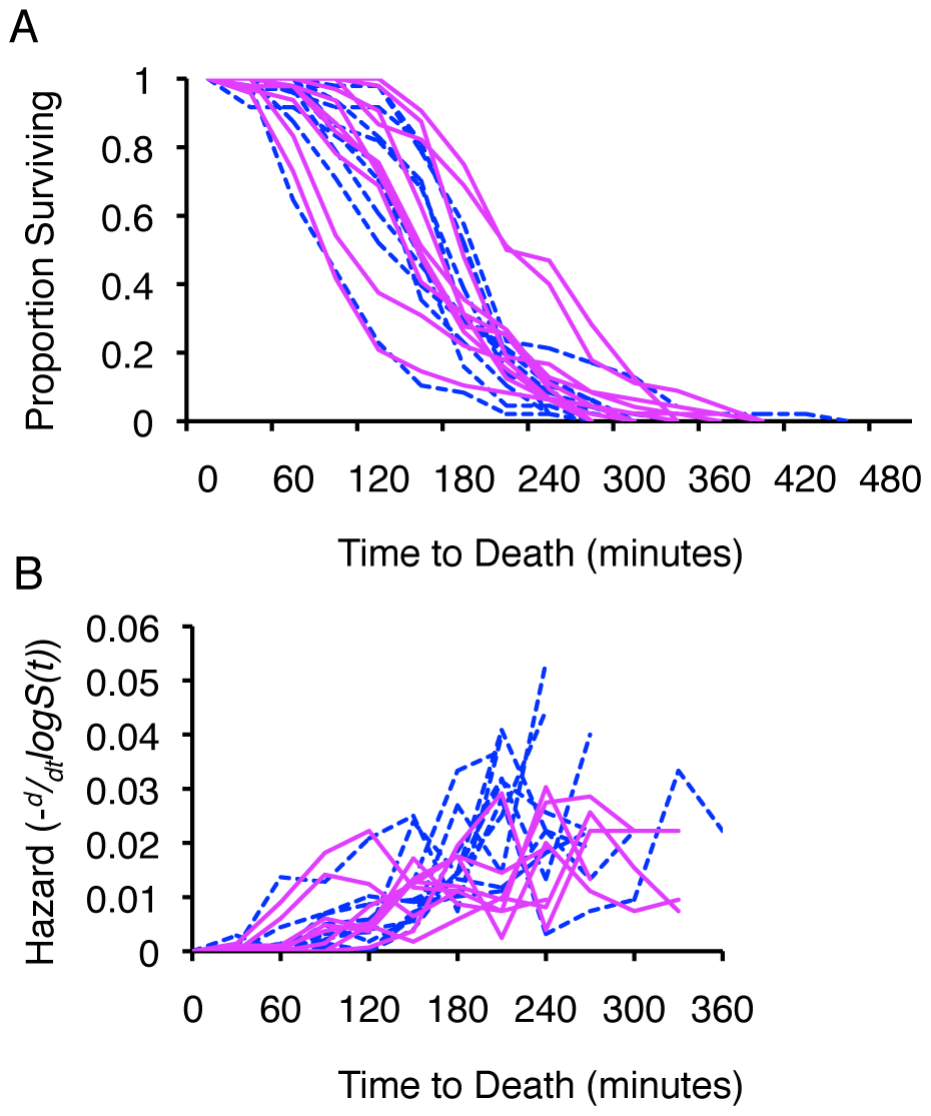
*C.* remanei survival during exposure to 3.5 mM H_2_O_2_, plotted by sire. (A) Age-specific survival (*S(t)*) and (B) age-specific hazard for males (dashed lines) and females (solid lines). Max hazard (100% risk of death within the next 30 minutes) for these data is 0.08. Control worms (housed in S basal only, not exposed to H_2_O_2_) experienced no death during the assay; mortality not shown. Heritabilities were high for females and males (*h^2^* = 0.95 and 0.75 respectively, Table 1) and were significantly greater than heritability for longevity or UV stress (Mann-Whitney U, P<0.0001).

Likewise, the ability to withstand oxidative stress was significantly different among the offspring of different sires (Cox proportional hazard; Females 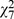  = 85.87, *P<*0.0001; Males 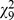 = 36.54, *P<*0.0001; Figure 3; Table S3). Heritability for oxidative shock resistance was the highest of the traits measured in our study; in females it was two times greater than that for heat stress resistance and more than 11 times greater than that for lifespan (Mann-Whitney U *P*<0.0001, Table 1). *CV_A_* estimates for oxidative and heat stress resistances were comparable.

**Figure 4:**
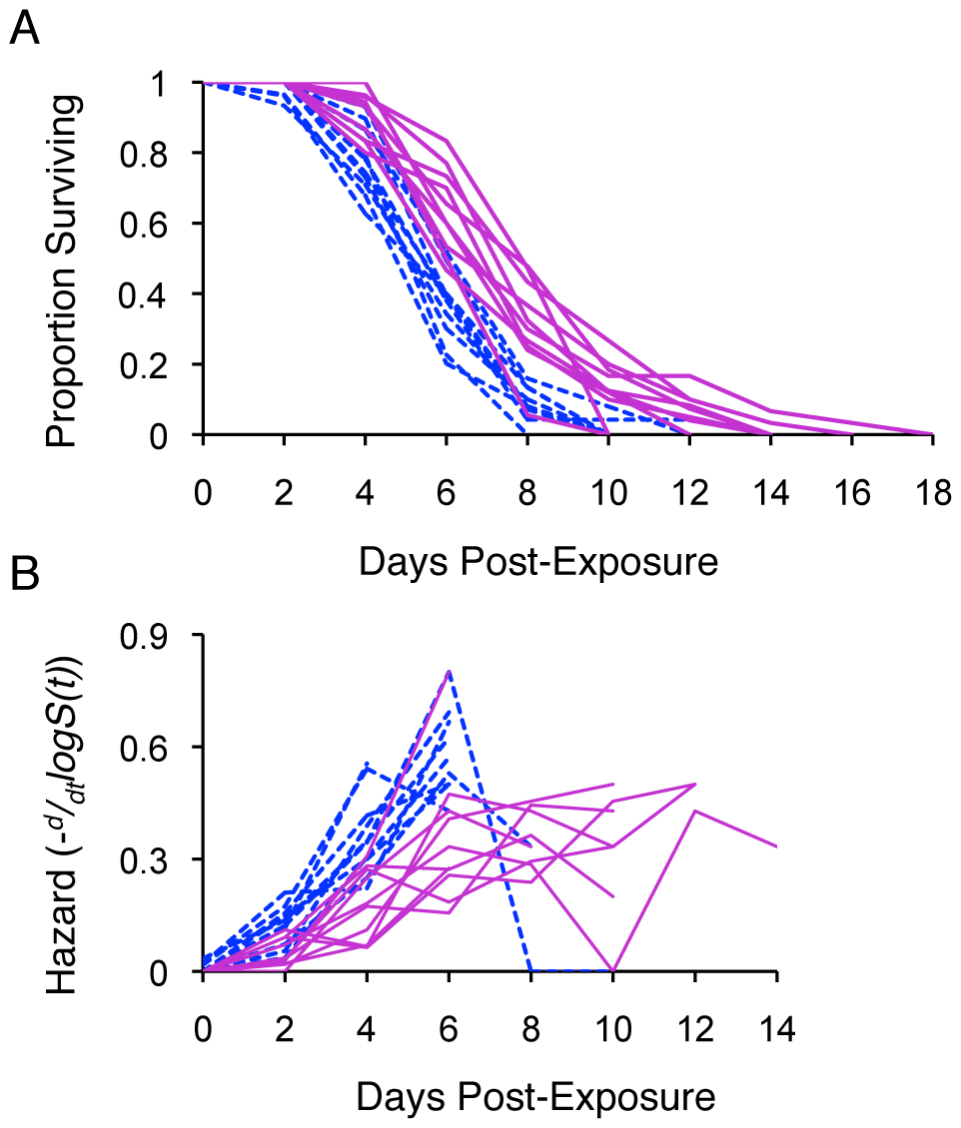
Mortality rates (hazard) after exposure of *C.* remanei to 2000 J/m^2^ UV, plotted by sire. Dashed lines = males, solid lines = females. (A) Age-specific survival (*S(t)*); (B) Age-specific hazard. Max hazard (100% risk of death within the next 30 minutes) for these data is 1.0. Although there is a large sex effect on oxidative stress resistance, there is no evidence for significant variation among offspring of different sires (*h* ^2^∼0.00, Table 1).

The ability to withstand UV stress (days survived after exposure to 2000 J/m^2^) was not significantly different among offspring of different sires (Females *F_9_* = 1.33, *P* = 0.22; Males = *F_9_* = 1.71, *P* = 0.09). Despite some differences in age-specific mortality (Cox proportional hazard; Females 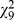 = 16.41, *P* = 0.06; Males 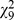 = 23.89, *P* = 0.04; Figure 4), we found no additive genetic variance for resistance to UV exposure, and heritability for the trait was likewise zero (Table 1; Table S4). Mean survival after UV exposure was significantly different between males and females (*F_1,460_* = 99.84, *P*<0.0001) as was age-specific mortality (Cox proportional hazard; 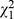 = 51.50, *P*<0.0001, Figure 4) with females living surviving longer on average (Females 6.72±0.18 days; Males 4.98±0.12 days). Males were less stress resistant to UV stress than were females.

## Discussion

### Contrasting patterns of natural genetic variation for longevity and stress resistance

We have shown that *C. remanei* has heritable genetic variation for lifespan (significant for females), and high heritability for two stressors hypothesized to be linked to longevity: heat and oxidative stress resistance. Heat, oxidative and UV stress resistance are all traits previously correlated with longevity in Age mutants [26]. Of the three, oxidative stress resistance has received the most attention in the aging literature, being a hypothesized mechanism for senescence. The oxidative stress hypothesis proposes that aging occurs because of the gradual accumulation of molecular and cellular damage caused by chemically reactive molecules, reactive oxygen species in particular [27]. A corollary of this hypothesis is that oxidative stress resistance ability, through preventative or reparative means, is a primary determinant of lifespan. Our data show dramatically different scales of genetic variation for longevity than for oxidative stress resistance. Although heritability and *CV_A_* for longevity were low in this population, they are non-zero, and similar to published estimates for other species [6], [28]. Heritability for oxidative stress response in this population of *C. remanei* was more than 11-fold that for longevity (Females, Table 1).

The difference in heritability estimates between longevity and oxidative stress resistance in our study likely reflects the complexity of the genetic relationship between the two traits. The oxidative stress theory of aging is coming under increasingly critical examination [29]. Most Age mutants are known to confer resistance to oxidative stress, *e.g. C. elegans daf-2* [30], and *D. melanogaster mth* [31]. Yet, genetic or genomic manipulations attempting to reduce oxidative damage, such as over-expression of antioxidants, have either been shown to increase lifespan [32], do nothing [33], or actually reduce lifespan [34]. Reductions of oxidative defenses, such as deletions of stress resistance genes, can result in increased oxidative damage and decreased lifespan [35], but sometimes have no effect [29], [36], or have *extended* lifespan in some cases [37]. Lastly, although some populations of invertebrates artificially selected for long life are oxidatively resistant [6], not all oxidatively resistant populations are long lived [38]. To some extent, the difference in heritability between these traits can be attributed to the large environmental variance typically observed for longevity, which appears in the denominator of heritability. However, a two-fold difference in genetic variation is still observed when the coefficient of additive genetic variation is use as the measure genetic variation instead of heritability ([24]; Table 1). Overall then, it is becoming clear that, while it may play a significant role in age-related disease, the relationship between oxidative stress resistance and longevity is complex.

### Stress-specificity of cellular stress responses

Although UV stress resistance is typically correlated with both extended lifespan and oxidative stress resistance in Age mutants [26], we found no genetic variation and thus no heritability for it in our study population. Our results raise the possibility that, in at least one rearing environment and genetic background, UV and oxidative stress resistance are genetically uncorrelated. This is particularly intriguing since there is a large body of literature demonstrating that cellular responses to specific stressors (*e.g.* pathogens, and oxidative, starvation and heat stress) use a limited number of common cellular pathways (IIS, TGF-ß, MAPK, JUN-K, HSF-1) and that those cellular responses are controlled through a likewise limited number of transcription factors (SKN-1, DAF-16, HSF-1, HIF-1, PHA-4). Indeed, the cellular response to any single stressor appears to be achieved through pathways at least partially overlapping cellular responses to other stressors [8].

### Environmental context of expressed variation

One possible reason for the difference in heritability estimates for longevity and oxidative stress resistance might be that longevity was measured in an artificially benign environment with regard to oxidative challenge. In a more naturalistic spatial and nutritional environment, oxidative stress resistance may indeed determine lifespan. Empirical studies have measured the heritabilities of traits in both stressful and non-stressful environments and found that heritabilities of some traits are higher under stressful conditions [29]–[41]. However, a relatively equal number of findings have been of the opposite effect: that heritability of traits is lower in stressful environs than in benign ones [40]–[42]. In our study, mortality under oxidative stress was 100% within eight hours of exposure. Because oxidative stress resistance and lifespan are inexorably linked in this experiment, it could be said that our stress resistance assays were in fact longevity assays under stressful conditions. Regardless of the relationship between levels of stress and levels of heritability for a particular trait, we can conclude that, for this population of *C. remanei*, lifespan under relatively benign conditions (perhaps most relevant to current human lifespan) is not determined by the same suite of genes as is resistance to oxidative stress in the environmental context of this study.

Overall, this study demonstrates that there is significant genetic variation for stress-related phenotypes within *C. remanei*, but that the relationship between stress resistance and longevity must be complex and environmentally contingent, if it exists at all. This illustrates that stress and longevity effects observed via mutagenesis may not always be reflected in natural variation; understanding the molecular basis of that variation should be highly informative of molecular mechanisms of longevity in its own right. *C. remanei* should provide a powerful system for such analyses.

## Supporting Information

Figure S1 **Mean survival time (in days) of** ***C. remanei*** **daughters regressed upon lifespan of mothers.** All worms were mated to three male *C. remanei* for 24 hours, and kept under typical laboratory conditions (20C, nematode growth media, lawn of *E. coli*).
(PDF)

Table S1 **Lifespan data (in days) for virgin *C. remanei* under typical laboratory conditions.** (20C, nematode growth media, lawn of *E. coli*).
(PDF)

Table S2 **Percent survival of female *C. remanei* exposed to 35.5°C for 16 hrs.** We used 60 one-day-old virgin adults per trial.
(PDF)

Table S3 **Mean survival time (in minutes) of half-sib families of *C. remanei* exposed to 3.5 mM H_2_O_2_ until death. N is number of offspring tested.**
(PDF)

Table S4 **Mean survival time (in days) of half-sib families of *C. remanei* exposed to 2000 J/m^2^ ultraviolet irradiation.**
(PDF)
