# Supplementary material for: Natural Variation for Lifespan and Stress Response in the Nematode Caenorhabditis remanei
Source: PLoS One. 2013 Apr 26;8(4):e58212. doi: 10.1371/journal.pone.0058212 (PMC3637273; doi:10.1371/journal.pone.0058212)

Figure S1. Mean survival time (in days) of *C. remanei* daughters regressed upon lifespan of mothers. All worms were mated to three male *C. remanei* for 24 hours, and kept under typical laboratory conditions (20C, nematode growth media, lawn of *E. coli*). Slope = 0.03.

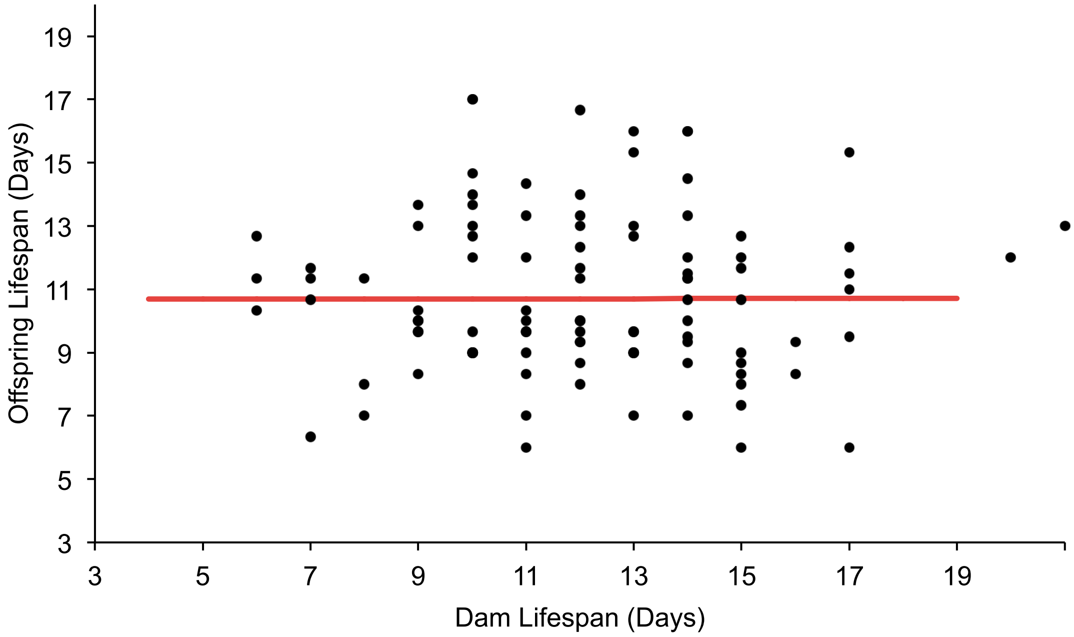

Supplement: Figure S1 — Mean survival time (in days) of C. remanei daughters regressed upon lifespan of mothers. All worms were mated to three male C. remanei for 24 hours, and kept under typical laboratory conditions (20C, nematode growth media, lawn of E. coli). (PDF) [file pone.0058212.s001.pdf]
