# Supplementary material for: Natural Variation for Lifespan and Stress Response in the Nematode Caenorhabditis remanei
Source: PLoS One. 2013 Apr 26;8(4):e58212. doi: 10.1371/journal.pone.0058212 (PMC3637273; doi:10.1371/journal.pone.0058212)
Supplement: Table S1 — Lifespan data (in days) for virgin C. remanei under typical laboratory conditions. (20C, nematode growth media, lawn of E. coli). (PDF) [file pone.0058212.s002.pdf]

Table S1. Lifespan data (in days) for virgin *C. remanei* under typical laboratory conditions (20C, nematode growth media, lawn of *E. coli*). N represents number of individuals. Stderr is standard error.

Sex	*Line*	*N*	*N Censored*	*Mean*	*Stderr*	*Median*	*Max. Observed Death (not censored)*
Female							
	PB234	29	10	15.33	1.41	13	29
	PB237	28	6	9.69	0.78	10	18
	PB241	27	1	12.05	1.07	12	25
	PB244	26	3	10.65	1.29	8	27
	PB245	38	6	13.53	0.70	14	23
	PB261	33	6	12.66	0.65	12	20
	PB266	24	10	12.03	0.87	11	22
	PB269	22	7	10.98	0.90	10	20
	PB271	43	2	12.35	0.97	13	25
	PB272	32	2	9.90	0.73	11	20
Male							
	PB237	24	6	15.53[a]	1.16	15	36
	PB241	26	10	17.05	1.39	16	37
	PB261	19	11	15.62	1.36	16	26
	PB266	17	9	16.79	1.50	17	31
	PB269	15	16	16.41[a]	1.49	15	25
	PB272	12	14	14.36[a]	1.39	13	21

[a]Mean survival time and its standard error were underestimated due to the largest observation being sensored. Estimation was restricted to the largest observed death (non-censored).
