# Supplementary material for: Natural Variation for Lifespan and Stress Response in the Nematode Caenorhabditis remanei
Source: PLoS One. 2013 Apr 26;8(4):e58212. doi: 10.1371/journal.pone.0058212 (PMC3637273; doi:10.1371/journal.pone.0058212)
Supplement: Table S2 — Percent survival of female C. remanei exposed to 35.5°C for 16 hrs. We used 60 one-day-old virgin adults per trial. (PDF) [file pone.0058212.s003.pdf]

Table S2. Percent survival of female *C. remanei* exposed to 35.5°C for 16hrs. We used 60 one-day-old virgin adults per trial. Means are least square means back-transformed from arcsin values. Stderr is standard error.

Line	N Trials	Mean % Survival	Stderr	Min % Survival	Max % Survival
PB237	8	67.40	+4.72/-5.01	51.28	80.33
PB244	6	52.91	+7.50/-7.94	41.67	96.43
PB245	8	42.00	+5.94/-6.12	8.77	71.12
PB258	10	78.75	+4.08/-4.46	46.03	98.39
PB269	10	79.62	+3.73/-4.06	61.67	91.53
PB271	10	33.23	+5.74/-5.86	3.28	60.66
PB272	10	64.00	+4.98/-5.26	37.70	75.00
