# Supplementary material for: Natural Variation for Lifespan and Stress Response in the Nematode Caenorhabditis remanei
Source: PLoS One. 2013 Apr 26;8(4):e58212. doi: 10.1371/journal.pone.0058212 (PMC3637273; doi:10.1371/journal.pone.0058212)
Supplement: Table S3 — Mean survival time (in minutes) of half-sib families of C. remanei exposed to 3.5 mM H2O2 until death. N is number of offspring tested. (PDF) [file pone.0058212.s004.pdf]

Table S3. Mean survival time (in minutes) of half-sib families of *C. remanei* exposed to 3.5mM H_2O_2 until death. N is number of offspring tested. Stderr is standard error.

Sire ID	Dam ID	N Daughters	Daughter Mean (Stderr)	N Sons	Son Mean (Stderr)
51	511	13	246.92 (18.34)	16	187.50 (5.81)
	512	16	215.63 (19.02)	16	178.13 (8.86)
	515	16	234.37 (18.62)	16	223.12 (11.28)
52	521	16	172.50 (7.98)[a]	12	140.00 (12.97)
	524	5	144.00 (32.03)[a]	16	200.63 (10.51)
	525	16	187.50 (12.09)[a]	16	189.37 (13.05)
61	612	16	116.25 (14.72)	16	108.75 (13.38)
	613	16	146.25 (17.51)	16	82.50 (6.98)
	615	16	73.13 (5.46)	16	125.63 (14.55)
62	621	16	93.75 (12.51)	16	140.62 (17.69)
	622	15	176.52 (19.69)	16	193.12 (16.65)
	625	16	144.38 (16.25)	16	127.50 (6.25)[b]
73	732	16	185.62 (14.02)	12	157.50 (16.15)
	733	15	185.63 (14.32)[b]	17	141.18 (11.14)
	734	15	144.37 (13.51)[b]	15	174.00 (10.23)
74	741	15	198.75 (21.42)[b]	16	159.38 (14.16)
	744	16	174.38 (16.71)	16	176.25 (6.64)
	745	16	178.12 (20.12)	15	204.00 (8.38)
83	832	16	221.25 (16.17)[a]	16	210.00 (11.94)
	834	5	186.00 (41.79)[a]	16	168.75 (14.46)
	835	16	260.62 (11.20)[a]	16	200.63 (8.97)
84	841	16	193.12 (10.24)	16	191.25 (11.90)
	842	16	241.88 (14.61)	16	275.62 (19.60)
	843	16	181.88 (6.96)	15	174.00 (5.24)
91	911	8	176.25 (31.28)	8	187.50 (19.43)
	912	8	146.25 (10.51)	9	203.33 (24.89)
	913	16	165.00 (16.66)	16	131.25 (13.93)
93	931	16	146.25 (17.29)	16	127.50 (13.55)
	932	16	198.75 (13.93)	16	161.25 (12.21)
	933	13	186.92 (18.34)	16	172.50 (14.62)

[a]Data not used in calculations of heritability or variance due to low replication of offspring from one or more dams.

[b]Mean survival time and its standard error were underestimated due to the largest observation being sensored. Estimation was restricted to the largest observed death by H_2O_2 (non-censored).
