# Supplementary material for: Natural Variation for Lifespan and Stress Response in the Nematode Caenorhabditis remanei
Source: PLoS One. 2013 Apr 26;8(4):e58212. doi: 10.1371/journal.pone.0058212 (PMC3637273; doi:10.1371/journal.pone.0058212)
Supplement: Table S4 — Mean survival time (in days) of half-sib families of C. remanei exposed to 2000 J/m2 ultraviolet irradiation. (PDF) [file pone.0058212.s005.pdf]

Supplementary Table 4. Mean survival time (in days) of half-sib families of *C. remanei* exposed to $2000 J/m^2$ ultraviolet irradiation. N is number of offspring tested. Stderr is standard error.

Sire ID	Dam ID	N Daughters	Daughter Mean (Stderr)	N Sons	Son Mean (Stderr)
12	121	10	5.8 (0.47)	8	4.25 (0.54)
	122	10	7.26 (0.57)	10	5.46 (0.68)
	123	7	8.83 (1.22)	8	3.94 (0.78)
13	131	9	7.03 (0.52)	10	6.14 (0.40)
	132	9	7.49 (0.58)	10	3.75 (0.34)
	135	6	6.17 (0.95)	10	3.29 (0.41)[b]
22	222	10	4.90 (1.06)	10	5.25 (0.51)
	224	10	7.00 (1.15)	10	6.01 (0.57)
	225	10	6.20 (0.63)	10	5.29 (0.51)
23	232	10	6.20 (0.81)	9	3.22 (0.36)
	233	10	6.67 (0.58)	10	5.33 (0.33)
	235	2[a]	4.50 (2.50)[a]	10	6.68 (1.04)
31	314	10	5.40 (0.48)	10	4.90 (0.78)
	315	8	5.50 (0.50)	10	5.18 (0.57)
34	342	10	7.60 (0.87)	10	4.87 (0.57)
	343	10	5.10 (0.71)	10	4.84 (0.39)
	345	5[a]	6.80 (1.56)[a]	10	4.40 (0.43)
42	422	9	6.91 (0.75)	10	4.90 (0.35)[b]
	424	10	7.00 (1.04)	10	6.40 (0.64)
	425	10	7.40 (0.88)	10	4.89 (0.43)[b]
44	442	10	7.70 (1.21)	10	5.89 (0.45)
	444	10	6.90 (0.91)	10	4.10 (0.50)
	445	10	7.70 (1.04)	10	5.22 (0.49)
51	513	10	9.20 (1.36)	10	4.82 (0.78)
	514	10	5.00 (0.45)	9	5.65 (0.56)
	515	10	6.50 (1.13)	10	3.40 (0.40)
52	522	10	7.20 (1.10)	8	4.00 (0.60)
	523	10	5.60 (0.56)	9	4.79 (0.71)
	524	10	7.90 (1.09)	10	5.38 (0.63)

[a]Data not used in calculations of heritability or variance due to low replication of offspring from one or more dams.

[b]Mean survival time and its standard error were underestimated due to the largest observation being sensored. Estimation was restricted to the largest observed death (non-censored).
